# Permeability and Porosity Development during the Carbonization of Coals of Different Coking Pressures

**DOI:** 10.1021/acs.energyfuels.0c04219

**Published:** 2021-03-15

**Authors:** M. Dolores Casal, Elvira Díaz-Faes, Carmen Barriocanal

**Affiliations:** Instituto de Ciencia y Tecnología del Carbono, INCAR-CSIC, Francisco Pintado Fe, 26, 33011 Oviedo, Spain

## Abstract

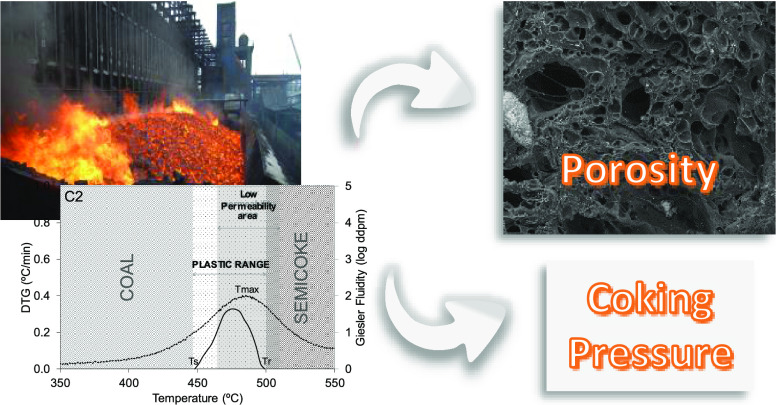

To obtain a better understanding of the development of coking pressure during the carbonization process, the plastic and semicoke layers of nine coking coals were investigated. The permeability of the plastic layer to the passage of gas and the porosity of the semicoke were analyzed at two temperatures, 500 and 800 °C. In the case of dangerous coals, there was a wide zone of low permeability covering most of the plastic layer and part of the semicoke, whereas safe coals had a very narrow permeability zone that affected only a small part of the plastic layer. It seems that dangerous coals have a higher porosity and a lower Hg apparent density than safe coals. In addition, the semicokes obtained at 800 °C from the dangerous coals had a higher macropore volume with pore size between 50 nm and 12 μm but a lower suprapore volume (pore size between 12 and 250 μm).

## 1 Introduction

Coking in industrial ovens produces swelling of coal mass that can generate excessive coking pressure if there is insufficient contraction at the end of the process. This phenomenon can cause operational problems due to difficulties in pushing operations that may cause damage to the oven walls and consequently substantial financial loss.^1−4^

During recent years, many papers have been published on the mechanism of coking pressure generation to evaluate the influence of different factors that contribute to the generation of excessive coking pressure during the coking of coals.^4−9^ Dangerous coals are included in coking blends because they usually produce high yield and high-quality coke. Coking pressure depends mainly on factors intrinsic to coal, although operational factors such as bulk density and heating rate are also important. One of the most important factors for the development of coking pressure is related to the thermoplastic properties of coals, i.e., its viscosity, and the permeability of the plastic mass, the structure of the semicoke in the coal plastic stage, and the amount of inerts in the coal are also important.^3,5−11^

In industrial coke ovens, where heat travels from the walls toward the center of the charge, there is a temperature gradient that leads to the coexistence of layers of different materials, i.e., coke, semicoke, coal in its plastic state, a mixture of dry coal with recondensed tar, and wet coal in the center of the oven. The plastic layer is a highly heterogeneous coal stage, where both chemical and physical processes that are of great importance for the development of the porous structure and the anisotropic development of the coke occur.^1^

Previous studies^12,13^ based on experiments carried out in a laboratory oven report the existence of two low-permeability layers on either side of the plastic layer. These studies suggest that all coking coals form a low-permeability layer on the coal side of the plastic layer, as a result of which gas is forced to exit initially via the coke side. The low-permeability layer consists of a mixture of coal with tar distilled from higher-temperature layers in the oven. As carbonization progresses, the plastic layer approaches the center of the charge and eventually disappears. It is suggested that it is the low-permeability layer on the semicoke side that determines coking pressure.^14,15^ However, it will also depend on the amount of fissuring and the pore structure of the semicoke. A number of works have emphasized the importance of the semicoke structure for the generation of coking pressure. Using X-rays, Zubkova showed that a displacement of the nonvolatiles mass of the coal charge takes place, causing a change in the solid residues.^16^ Duffy et al.^6^ proposed a pore coalescence mechanism, whereby internal gas pressure is released from the coal charge, preventing the high oven wall pressures from increasing. More recently, semicokes were also studied using micro-CT analysis to understand the pore structure.^10^ In general, papers focused on porosity suggest that the porosity structures of semicokes from dangerous and safe coals are different. However, they were not focused on the impact of large-size pores.^6,10^

Accordingly, the main aim of the present study is to correlate the variation in permeability during the carbonization process and the porous structure of semicokes produced at various temperatures with the coking pressure and contraction of coals of various ranks. Pores of size up to 250 μm were measured to establish the influence of large pores on the coking pressure.

## 2 Experimental Section

### 2.1 Materials

Nine coals of different ranks were selected from those commonly used in the cokemaking industry. Proximate analyses were performed following the ISO562 and ISO1171 standard procedures for volatile matter (VM) and ash content, respectively. The elemental composition was measured using LECO CHNS-932 and LECO VTF900 instruments for C, H, N, S, and direct oxygen determination, respectively.

### 2.2 Thermogravimetric Analysis (TG/DTG)

The TG/DTG analysis of the coals was carried out using a TA Instruments SDT 2960 thermoanalyzer. Samples (10–15 mg) with particle sizes of <0.212 mm were heated up to 1000 °C at a rate of 3 °C/min under a nitrogen flow of 100 mL/min. From the data obtained, the volatile matter evolved up to a specific temperature (VMT) and the derivative of the weight loss curve (DTG curve) was calculated. The volatile matter evolved in a specific temperature range was calculated as the difference between the volatile matter evolved up to two specified temperatures (VM*T*_1_–*T*_2_). In addition, *T*_max_, the temperature of maximum volatile matter evolution, was derived from the TG/DTG curves.

### 2.3 Thermoplastic Properties

The thermoplastic properties of the coals were tested by the Gieseler method in an R.B. Automazione Gieseler plastometer PL2000, following the ASTM D2639-74 standard procedure. The parameters derived from this test were: (i) softening temperature, *T*_s_; (ii) temperature of maximum fluidity, *T*_f_; (iii) resolidification temperature, *T*_r_; (iv) plastic range, *T*_r_ – *T*_s_, which is defined as the difference between the resolidification and softening temperatures; and (v) maximum fluidity, MF, expressed as dial divisions per minute (ddpm).

### 2.4 Plastic Layer Permeability

A representative coal sample of 2 g ground to <3 mm was placed in a cylindrical quartz tube of 20 mm internal diameter. The height of the coal bed was 10 mm. A layer of alumina was placed at the top and bottom of the coal bed to ensure uniform heating and to prevent the scattering of fine particles. The coal samples were heated up to 800 °C at a heating rate of 3 °C/min, and nitrogen was introduced from the bottom of the coal layer at a flow rate of 0.01 m/s following a previously used procedure.^14^ Tests were carried out at least twice.

### 2.5 Semicoke Preparation

The semicokes were prepared in a sole-heated oven using 80 g of sample ground to <1 mm with a bulk density of 820 kg/m^3^. For all of the coals, two semicokes were prepared, one at resolidification temperature (ca. 500 °C) and the other one at 800 °C to observe the evolution of the porous structure with temperature. The first set was labeled SK500, and the second set was labeled SK800. The temperature on the sole was fixed at 600 and 900 °C, and the coals were maintained for 2 h in the oven to obtain the semicokes SK500 and SK800, respectively. The temperatures on the sole and on the top of the charge were monitored to obtain the desired temperatures. Finally, the sample was taken out of the oven to cool down.

### 2.6 Textural Characterization

The true density (ρHe) of the semicokes was measured by means of helium pycnometry in a Micromeritics Accupyc 1330 pycnometer. Their apparent density (ρHg) was determined using mercury in a Micromeritics autopore IV 9500 mercury porosimeter. The porosity and total pore volume were calculated by comparing the true and apparent densities. The pore size distribution was obtained by applying increasing pressure to the sample from 0.1 to 227 MPa to measure pore sizes in the range of 250 μm to 5.5 nm by means of the Washburn equation.^17^ The pore sizes were classified into three categories, i.e., suprapores (250 > dp > 12 μm), macropores (12 μm > dp > 50 nm), and mesopores (50 > dp > 5.5 nm). The micropore volume was calculated by difference and included all those of size dp < 5.5 nm. Cubic pieces of semicoke taken from the lower part of the semicoke with approximately 1 cm height were used for the apparent density and Hg porosimetry measurements (see Figure S1).

### 2.7 Morphological Characterization

Representative semicoke samples of the different coals and temperatures were analyzed by field emission scanning electron microscopy (FE-SEM). Images were acquired on a Quanta FEG 650 system (FEI company) operated at 25 kV.

### 2.8 Evaluation of Coal Dangerousness

Coking pressure was measured at the Centre Pyrolyse Marienau (CPM) in a 400 kg movable-wall oven, and the maximum pressure detected during the process was used to evaluate the dangerousness of the coals.^1,20^ The Koppers-INCAR test was also used to assess the dangerousness of the coals.^19^ Briefly, 80 g of a coal sample, ground to <1 mm size, was placed inside a stainless steel crucible and heated in a sole-heated oven up to 900 °C, for 2 h. The change in charge height during heating, compared to that of the initial height of the coal sample, was recorded on a graph in millimeters. Contraction is expressed in negative values, while positive values indicate expansion. The parameters derived from the curves are: (i) expansion, which is usually present in the case of dangerous coals and appears in the curves at the beginning of the test, and (ii) contraction/expansion, which is the difference between the initial height of the coal charge and the final height of the semicoke charge.

## 3 Results and Discussion

For this work, a wide range of bituminous coals, with a volatile matter content ranging between 16.7 and 33.4 wt %, were studied (Table 1). These coals are commonly used by the steel industry in blends to produce metallurgical coke.

**Table 1:** Main Characteristics of the Coals Studied

	dangerous coals							safe coals	
coal	C1	C2	C3	C4	C5	C6	C7	C8	C9
ash (wt % db)^a^	6.6	5.6	6.6	10.2	8.5	9.0	4.6	8.6	7.4
VM (wt % db)^a^	16.7	17.7	18.3	17.4	24.9	24.0	26.0	33.0	33.4
C (wt % db)^a^	84.0	85.6	83.9	79.8	79.9	80.2	85.4	77.9	79.1
H (wt % db)^a^	4.3	4.4	4.4	4.2	4.7	4.7	4.7	5.1	5.0
N (wt % db)^a^	1.4	1.6	1.5	2.8	1.3	2.2	1.1	2.2	1.5
S (wt % db)^a^	1.0	0.6	0.7	0.5	0.8	0.6	0.4	0.6	1.1
O (wt % db)^a^	2.4	3.6	2.6	4.0	4.9	4.9	3.9	6.7	6.1

a Dry basis.

**Figure 1:**
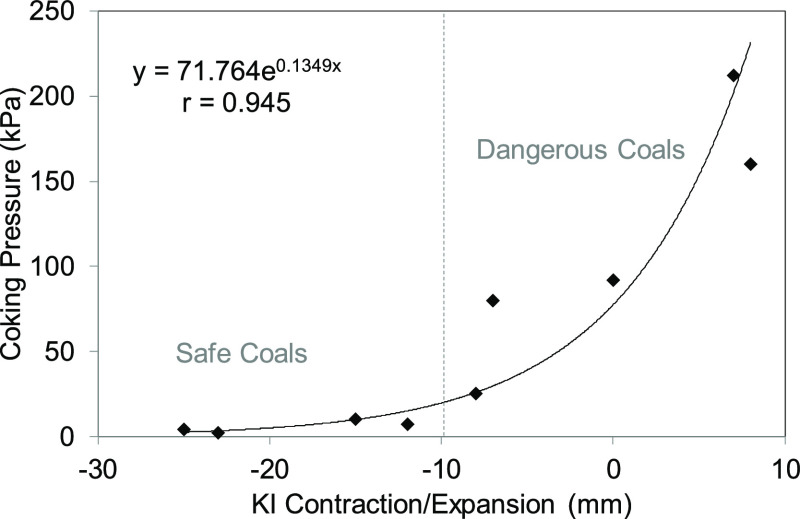
Relationship between coking pressure measured in a 400 kg oven and Koppers-INCAR contraction.

Table 2 shows the semicoke contraction/expansion data obtained from the Koppers-INCAR test and the coking pressure data obtained from the movable-wall oven. By comparing the data resulting from both methods, the trend obtained was adjusted to an exponential curve (Figure 1). Both methods allow a clear distinction to be made between safe and dangerous coking coals. A coal is considered dangerous during coking if (i) the Koppers-INCAR contraction is lower than 10 mm^19^ and (ii) the coking pressure is higher than 10–30 kPa.^1,20^ Therefore, coals C1–C4 are to be considered as dangerous, and C8 and C9 as safe coals. There are, however, three coals (C5, C6, and C7) that are close to the preestablished limits, and it is difficult to determine to what degree they are safe. These borderline cases may behave as dangerous or safe coals depending on the coking conditions used, i.e., bulk density, heating rate, particle size, flue temperature, etc.^1^

**Table 2:** Coking Pressure Measured in a 400 kg Movable-Wall Oven and Semicoke Contraction/Expansion Measured in the Koppers-INCAR Apparatus

	dangerous coals							safe coals	
	C1	C2	C3	C4	C5	C6	C7	C8	C9
Koppers-INCAR expansion (mm)	16	17	8	2	0	0	0	0	0
Koppers-INCAR contraction/expansion (mm)	7	8	0	–7	–8	–12	–15	–23	–25
coking pressure (kPa)	212	160	92	80	25	7	10	2	4

### 3.3 Gieseler Fluidity and Thermal Decomposition

Although no one single coal property has been linked to the generation of coking pressure, it is generally agreed that the fluidity/viscosity of the plastic layer plays a very important role.^6,8,10,14^ A comparison of the results of the Gieseler test (Table 3) with the expansion/contraction KI values and coking pressure data (Table 2) indicates that, in general, when the coking pressure increases and, consequently, KI contraction decreases, coal plasticity development proceeds at a higher temperature, the plastic range (*T*_r_ – *T*_s_) narrows, and coal fluidity decreases (Table 3). The only exception to these trends is provided by coal C5, which is situated in the limit of dangerousness that has a higher maximum fluidity and plastic range than C6 and C7.

**Table 3:** Parameters Derived from the Gieseler Test

		*T*_s_ (°C)^a^	*T*_f_ (°C)^b^	*T*_r_ (°C)^c^	*T*_r_ – *T*_s_ (°C)^d^	MF (ddpm)^e^
dangerous coals	C1	459	474	498	39	2
	C2	445	475	502	57	44
	C3	440	470	491	51	8
	C4	450	477	498	48	8
	C5	398	455	494	96	2712
	C6	417	458	494	77	672
	C7	414	452	486	72	631
safe coals	C8	393	438	472	79	3184
	C9	394	436	473	79	5061

a *T*_s_: softening temperature.

b *T*_f_: maximum fluidity temperature.

c *T*_r_: resolidification temperature.

d *T*_r_ – *T*_s_: plastic range.

e MF: maximum fluidity.

The thermogravimetric analysis of coals has been made taking into account the interdependence of devolatilization, fluidity, and coking pressure. The most important parameters derived from this analysis are summarized in Table 4. It can be seen that in the case of the dangerous coals, as the maximum rate of volatile matter evolution (DTG_max_) decreases, the temperature of maximum volatile matter evolution increases (*T*_max_), whereas the amount of volatile matter (VM) emitted decreases (higher coke yield).

**Table 4:** Parameters Derived from the Thermogravimetric Analysis

coal	VM_500_ (%)^a^	VM_400–500_ (%)^a^	VM_500–750_ (%)^a^	DTG_max_ (% min^–1^)^b^	*T*_max_ (°C)^c^	CY (%)^d^	*T*_r_ – *T*_max_ (°C)^e^
C1	43.6	9.1	42.4	0.352	483	80.9	15
C2	45.8	9.5	41.9	0.398	486	80.3	16
C3	46.1	10.8	35.5	0.404	477	78.3	14
C4	45.4	10.2	41.8	0.351	484	80.9	14
C5	60.8	18.4	29.9	0.567	467	74.4	23
C6	61.5	17.9	30.7	0.557	466	74.9	28
C7	62.8	20.1	29.6	0.629	461	73.2	25
C8	70.3	31.4	23.7	0.888	446	66.7	26
C9	73.3	31.9	21.0	0.852	446	67.3	27

a VM: volatile matter release up to a specific temperature or temperature interval and normalized to 100%.

b DTG_max_: rate of maximum volatile matter evolution.

c *T*_max_: temperature of maximum VM evolution.

d CY: coke yield.

e Difference between the resolidification temperature (*T*_r_, Gieseler test) and *T*_max_.

**Figure 2:**
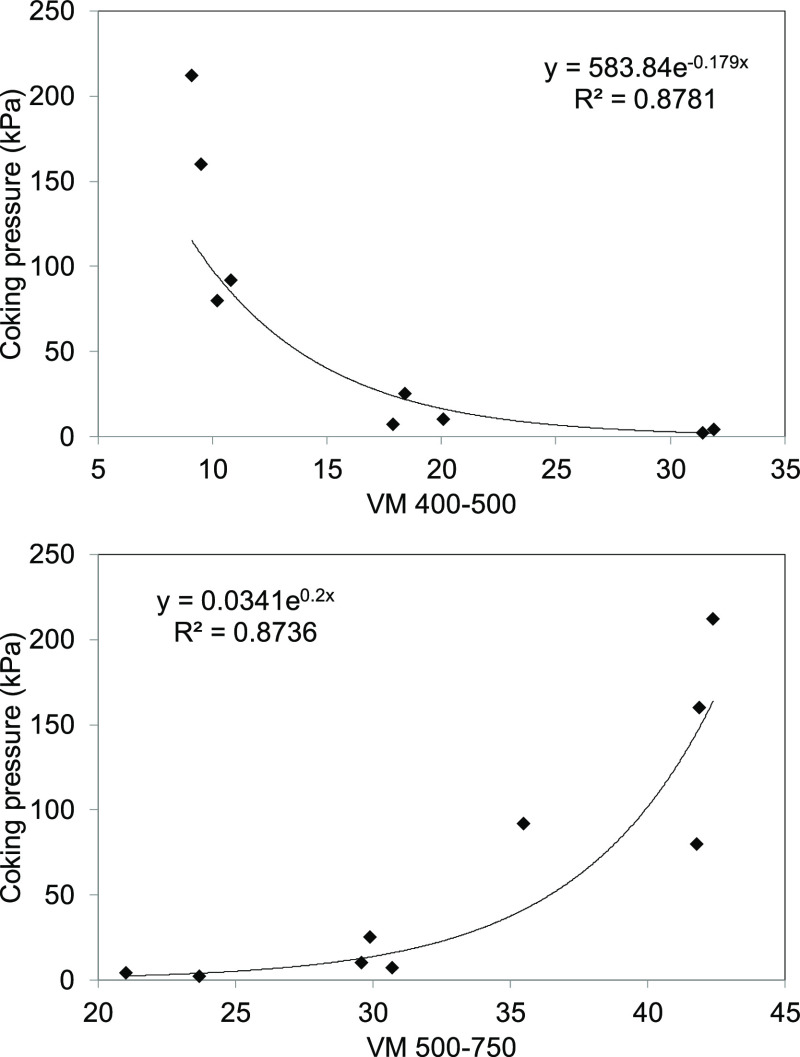
Relationship between coking pressure and the VM released during the plastic stage (MV 400–500 °C) and during the postplastic stage (500–750 °C).

In addition, the relationship between the release of volatile matter during the plastic (MV 400–500 °C) and postplastic stages (MV 500–750 °C) and the coking pressure is plotted in Figure 2. This shows that in the dangerous coals, the amount of VM evolved in the plastic stage is lower than in the safe coals, whereas the quantity of VM evolved in the postplastic stage is higher than it is in the safe coals. It seems that an abundance of high-molecular-weight compounds, VM, liberated between 500 and 750 °C, is associated with coals with a low KI contraction/expansion and a high coking pressure. These results agree with those of a previous study,^21^ which showed that the presence of heavy compounds in the primary tar was responsible for the high coking pressure.

Similar relationships, but with linear fittings were obtained using KI contraction/expansion instead of coking pressure. Correlation coefficients of *r*^2^ = 0.861 and 0.830 were obtained in the cases of VM_400–500_ and VM_500–750_, respectively.

**Figure 3:**
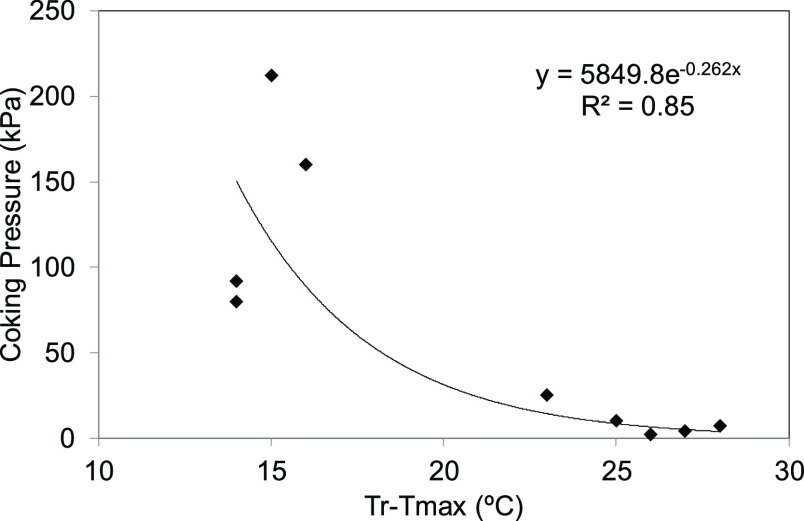
Relationship between coking pressure and the difference between the resolidification temperature (*T*_r_, Gieseler test) and the temperature of maximum evolution of volatile matter (*T*_max_, TGA).

Previous research works have established that there is a clear relationship between three phenomena common to the carbonization process,^18,22^ i.e., the development of plasticity, the release of VM, and the generation of coking pressure. Coal devolatilization and the plastic stage take place in similar temperature ranges. If the volatile matter evolves when the coal is in a highly fluid state, then it is easier for the volatiles to escape rather than to become trapped within the mass. On the other hand, if the volatiles evolve in a viscous medium, then it is more difficult for them to find their way out. In this study, the difference between *T*_r_ (resolidification temperature in the Gieseler test) and *T*_max_ (the temperature of maximum evolution of volatile matter) has been plotted against coking pressure (Figure 3 and Table 3). It can be seen that *T*_r_ – *T*_max_ shifts to lower values with the increasing dangerousness of the coals.

If *T*_r_ – *T*_max_ is low, most of the volatiles will evolve near the resolidification temperature and, consequently, in a very viscous medium. On the other hand, if *T*_r_ – *T*_max_ is high, the volatiles will evolve closer to the temperature of maximum fluidity, making it easier for them to escape. This explanation agrees with the previous results,^3,10,23,24^ where a coking pressure buildup mechanism was proposed, whereby below a certain temperature, it is not the amount of coal that decomposes that determines the development of coking pressure but the amount of volatiles evolved in the resolidification temperature range.

### 3.4 Permeability of the Plastic Coal Layer

Permeability test is a method that establishes the pressure drop (PD) needed to maintain a constant flow of gas through a coal layer during the pyrolysis process. It therefore indicates the degree of facility with which a gas traverses the coal plastic layer.

**Figure 4:**
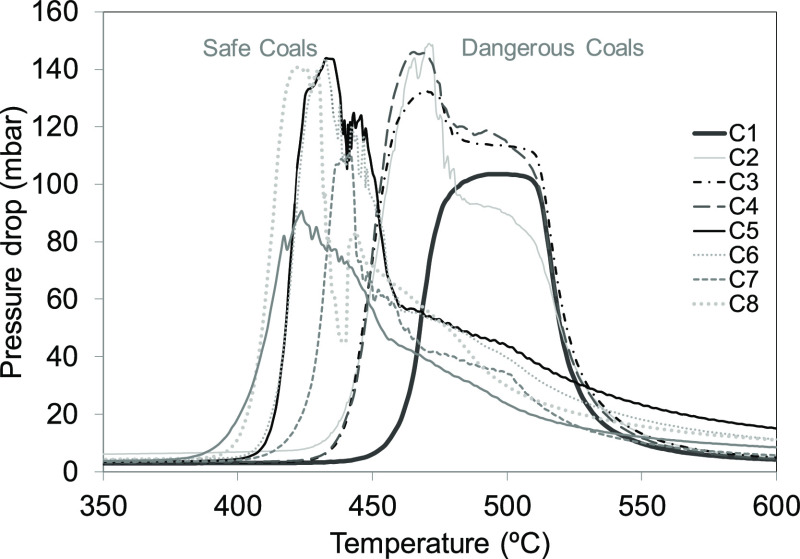
Changes in pressure drop in permeability test with temperature for all of the coals studied.

Changes in the pressure drop provided by the permeability test with temperature for all of the samples studied are shown in Figure 4. The main parameters obtained from the curves are presented in Table 5: the maximum pressure drop (PD_max_), the temperature at which the pressure drop (PD) reaches its maximum value, the temperature of the initial increase in PD, and the temperatures *T*_1_ and *T*_2_, which correspond to the beginning and end of the high-PD zone, respectively.

**Table 5:** Parameters Derived from the Permeability Test

coal	PD_max_ (mbar)^a^	TPD_max_ (°C)^b^	*T*_ini_ (°C)^c^	*T*_1_ (°C)^d^	*T*_2_ (°C)^e^	*T*_2_ – *T*_r_ (°C)^f^
C1	104	498	442	476	512	14
C2	149	471	423	463	507	5
C3	133	470	419	458	510	19
C4	146	467	419	462	507	9
C5	144	434	396	426	460	–34
C6	143	433	396	426	460	–34
C7	110	442	400	436	471	–15
C8	140	422	385	420	447	–25
C9	91	424	378	417	456	–17

a PD_max_: maximum pressure drop.

b TPD_max_: temperature at which the pressure drop reaches the maximum value.

c *T*_ini_: temperature of initial increase of PD.

d *T*_1_: temperature at the beginning of the high-PD zone.

e *T*_2_: temperature at the end of the high-PD zone;.

f Difference between *T*_2_ and resolidification temperature (*T*_r_, Gieseler).

It can be seen from Figure 4 that, as the temperature increases up to a certain value, the pressure drop begins to increase. Then, when it reaches a maximum value, it begins to decrease. It should also be noted that the zone of highest pressure drop is the low-permeability zone. The value of the maximum PD derived from our experiments cannot be used to differentiate dangerous from nondangerous coals. What appears to be important is not the absolute value of maximum PD but the temperature range in which permeability is low. This is reflected in the graph, where the difference between two groups corresponding to dangerous and safe coals is clearly observed. In the case of the dangerous coals, the pressure drop starts at higher temperatures, above 400 °C, and minimum permeability is reached at higher temperatures, above 460 °C.

In general, the maximum pressure drop range (*T*_2_–*T*_1_) is greater in the case of dangerous coals, which show a sharp reduction in pressure drop in the zone of *T*_2_, while the pressure drop in the safe coals proceeds at a more gradual rate.

An attempt has been made in this study to link the plasticity data from the Gieseler test, the maximum volatile matter emissions from the thermogravimetric analysis, and the parameters obtained from the permeability test.

**Figure 5:**
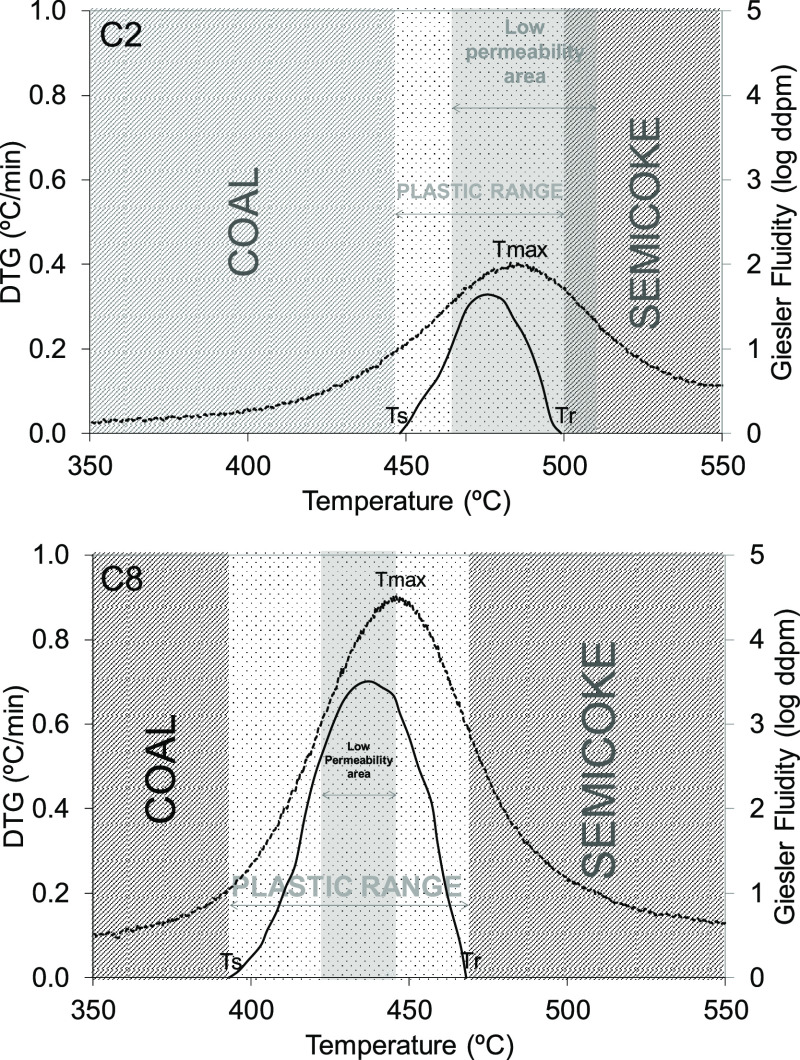
Location in a coking oven of the Gieseler fluidity area, volatile matter released as measured by the thermogravimetric test, and low-permeability zones for the dangerous coal C2 and safe coal C8.

In Figure 5, the data from the three different tests are superimposed, together with the different stages of the coal carbonization process (coal, plastic range, and semicoke). Data collected from the C2 coal, a dangerous coal, and C8, a safe coal, were plotted as examples of all of the coals studied. A comparison of the three sets of data collected for the dangerous coal clearly shows that the low-permeability zone (*T*_2_–*T*_1_) starts during the plastic stage and continues after the resolidification temperature, that is, *T*_r_ < *T*_2_, while in the case of safe coal, this area is much shorter and remains inside the plastic range. In general, *T*_2_–*T*_r_ is a positive value for dangerous coals, whereas it is negative for safe coals (Table 5).

As mentioned above, high-coking-pressure coals develop low plasticity and their maximum Gieseler fluidity occurs at higher temperatures than the safe coals. During the resolidification stage (*T*_r_ – *T*_f_), a mass with a high viscosity is generated, which makes it harder for gas to leave. Inside the oven, the low-permeability area corresponds to part of the plastic, postplastic, and semicoke areas (Figure 5). It is also important to bear in mind that the maximum amount of volatile matter will be released near the resolidification temperature (*T*_f_ < *T*_max_ < *T*_r_). As a result, the heavy organic compounds present in the exhaust gas stream will be unable to reach the coke side, the hottest part of the oven, and will move to the coldest part, the oven center (Figure 5). Once there, they will condense as coal tar.

Safe coals, on the other hand, form a high-fluidity material at lower temperatures. Their plastic stage therefore will be less viscous than in the case of dangerous coals. Their low-permeability zone will be narrower, start at a lower temperature, and will not exceed the resolidification temperature (*T*_r_ > *T*_2_). The maximum release of volatile matter in safe coals occurs in the temperature zone of maximum fluidity, and their components have a lower molecular weight than in the case of dangerous coals.^21^ When gas is released, the zone of low permeability comes to an end and the gas evolves without restriction. Due to the temperature gradient, the gas will move toward the hotter part of the oven, to where the already formed coke is. In this case, the gases will not condense on the cooler side of the plastic zone (Figure 5). These results agree with those of Koch et al.^25^ who reported that in the case of dangerous coals, volatile material migrates from the plastic layer to the cold side, whereas in the case of safe coals, migration is toward the hot side.

### 3.5 Relationship between the Porous Structure of the Semicoke and the Coking Pressure

The porous structure of semicoke depends on the processes that take place in the plastic stage. Gases evolved during the devolatilization of the coals form bubbles that move toward the boundaries of the grains, leaving voids behind.^7^ In addition, expanding gas may also cause the development of more porosity or the enlargement of existing pores. The viscosity characteristics of the plastic stage may therefore be critical for pore development.^6,10,26,27^ It is known that pore properties change between 500 and 800 °C;^16,18,28^ for this reason, the porous structures of the semicoke obtained at 500 and at 800 °C were characterized to obtain a better understanding of the development of coking pressure.

**Figure 6:**
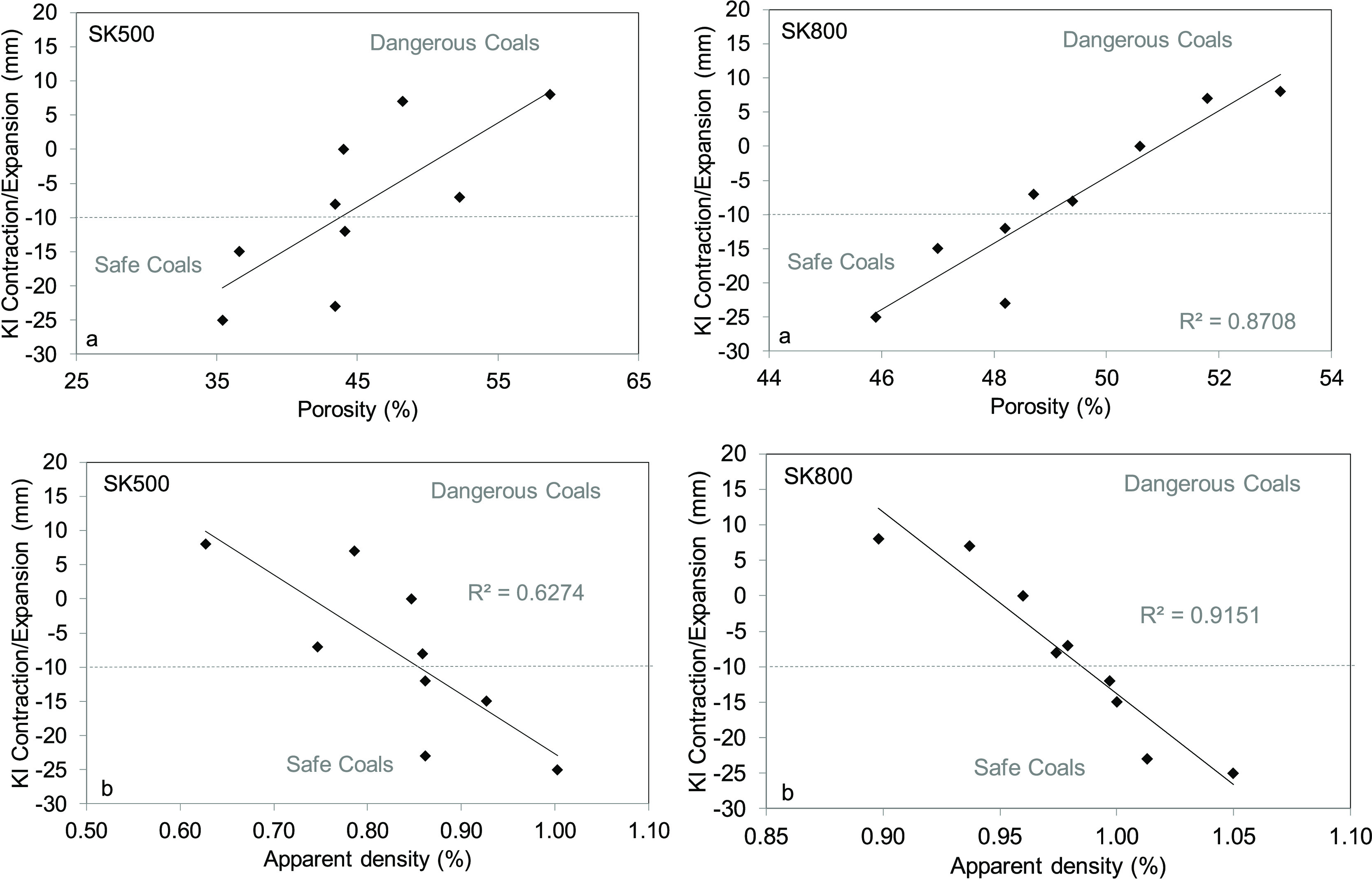
Relationship of (a) porosity and (b) apparent density of the semicokes produced at 500 and 800 °C with Koppers-INCAR contraction.

Table 6 shows the results of the measurements of the true and apparent densities, porosities, and pore volumes of all of the semicokes prepared. Both the apparent and true densities tend to increase with the increase in temperature. This increase indicates a compaction of the porous structure, and, in turn a reduction in the volume as a consequence of heating.^16,28^ Except for coals C2 and C4, the porosities at 500 °C are lower than those at 800 °C. Figure 6 shows the relationships between the total porosity and Hg apparent density of semicokes produced at 500 and 800 °C with Koppers-INCAR contraction. Similar relationships, but with exponential fittings, were obtained using coking pressure instead of the Koppers-INCAR contraction. Correlation coefficients of *r* = 0.706 and 0.831 were obtained in the case of porosity at 500 °C, and 0.732 and 0.866, respectively, in the case of SK800. These graphs are not included to avoid excessive length. The apparent density tends to be higher and porosity tends to be lower for the semicokes prepared with coals that show a high contraction and a low coking pressure.^10^

**Table 6:** Textural Characteristics of the Semicokes Prepared at 500 and 800 °C

semicoke	ρ_He_ (g/cm^3^)^a^	ρ_Hg_ (g/cm^3^)^b^	ε (vol %)^c^	*V*_tot_ (mm^3^/g)^d^
SK500				
C1	1.517	0.786	48.2	613
C2	1.518	0.627	58.7	937
C3	1.512	0.847	44.0	519
C4	1.565	0.747	52.3	700
C5	1.518	0.859	43.4	506
C6	1.542	0.862	44.1	512
C7	1.463	0.927	36.6	395
C8	1.523	0.862	43.4	503
C9	1.553	1.003	35.4	353
SK800				
C1	1.941	0.937	51.8	553
C2	1.914	0.898	53.1	592
C3	1.943	0.960	50.6	527
C4	1.911	0.979	48.7	498
C5	1.927	0.974	49.4	507
C6	1.926	0.997	48.2	484
C7	1.888	1.000	47.0	471
C8	1.956	1.013	48.2	476
C9	1.941	1.050	45.9	437

a Helium true density.

b Hg apparent density.

c Porosity.

d Total pore volume.

The amount of volatile matter released during the heating of dangerous coals is lower than that of typical safe coals. These volatiles are retained inside the resolidified structure, leading to an increase in volume with a consequent decrease in apparent density and an increase in porosity. In a previous study, it was also found that dangerous coals produced semicokes with a higher porosity, but only pores <12 μm were taken into account.^29^ In the present study, the results obtained include pore sizes of up to 250 μm. Other authors also found that the porosity of the cokes increased with the buildup of internal gas pressure measured in a double-wall heated oven. A possible explanation for this is that the collapse of pores that normally occurs before resolidification did not take place in cokes derived from coals with a high internal gas pressure.^10^

**Figure 7:**
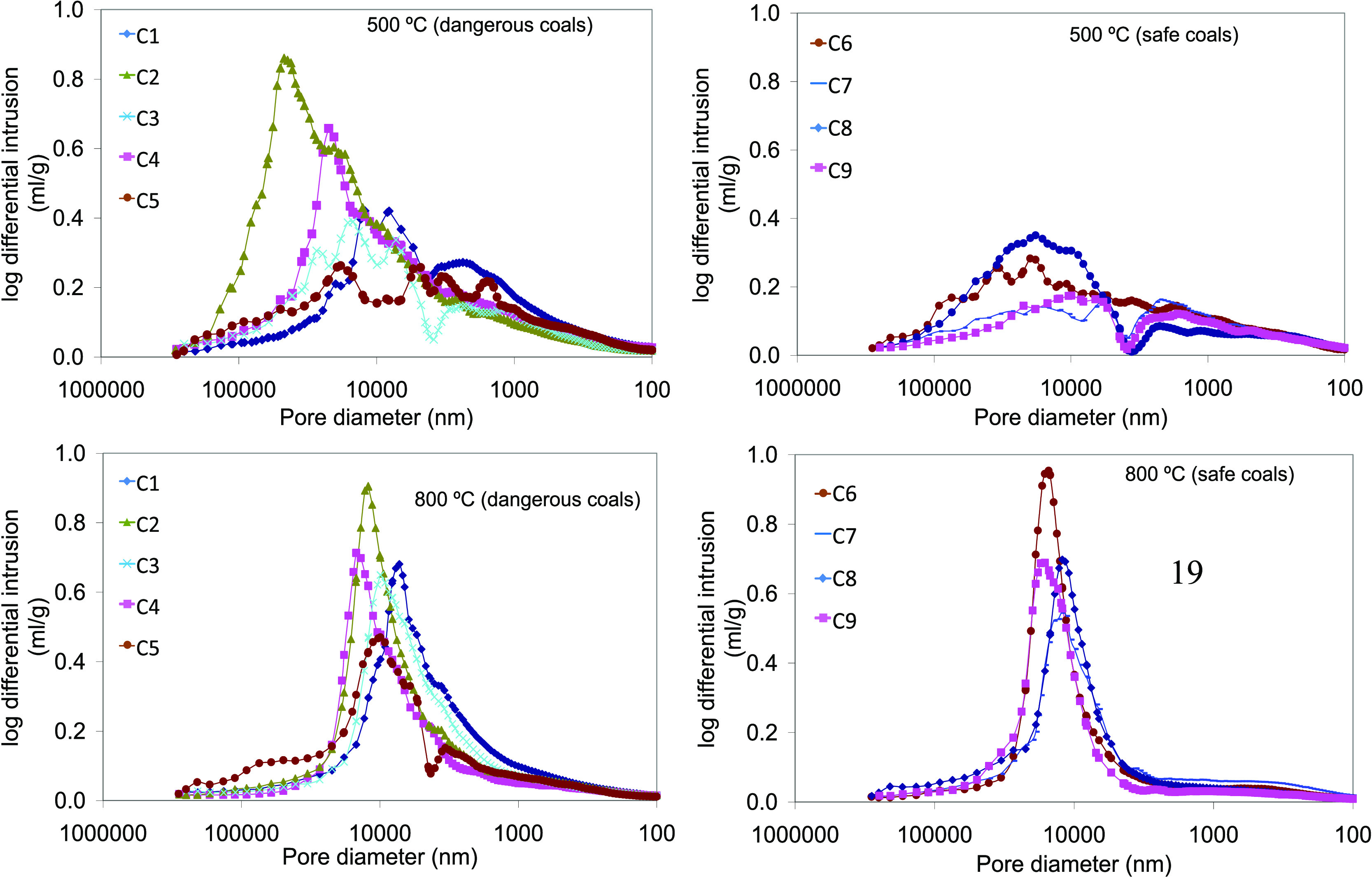
Pore size distributions measured by Hg porosimetry of the semicokes produced at 500 and 800 °C.

Figure 7 shows the differential intrusion curves for the semicokes obtained at 500 and 800 °C, which give an idea of the pore size distribution. The coals have been separated according to their coking pressure characteristics. From the curves, it is apparent that, while at 800 °C, the distribution is similar between dangerous and safe coals, at 500 °C, the volume corresponding to pore sizes between 10 and 100 μm is larger in dangerous coals, especially in the case of low-volatile coals. At 800 °C, all of the gas has escaped from the semicoke structure and the pore size distributions of the semicokes are much more similar than at lower temperatures.

The supra-, macro-, meso-, and micropore volumes of all of the semicokes were calculated from the mercury intrusion curves and are included in Table 7. The results show that the semicokes are macroporous materials, with a percentage of supra- and macropores higher than 80% of the total pore volume. The porosity is mostly dominated by macroporosity followed by supraporosity.

**Table 7:** Pore Size Distribution of the Semicokes Obtained at 500 and 800 °C

semicoke	*V*_supra_ (mm^3^/g)^a^	*V*_macro_ (mm^3^/g)^b^	*V*_meso_ (mm^3^/g)^c^	*V*_micro_ (mm^3^/g)^d^
SK500				
C1	129.2	400.6	25.7	57.1
C2	586.3	284.2	21.1	45.2
C3	142.3	290.9	19.2	66.4
C4	301.2	316.2	25.5	56.9
C5	175.0	274.7	20.6	35.3
C6	216.1	233.8	19.8	41.8
C7	117.2	204.2	25.9	47.4
C8	256.0	184.1	21.4	41.6
C9	101.1	184.2	19.0	48.3
SK800				
C1	80.8	402.2	8.4	61.1
C2	188.5	331.5	11.2	60.3
C3	87.5	371.2	10.3	57.6
C4	175.6	252.4	8.7	61.0
C5	166.3	261.4	9.9	69.7
C6	252.7	171.1	9.7	50.5
C7	154.9	228.3	8.7	78.7
C8	188.7	212.1	7.0	67.8
C9	237.9	134.5	6.8	58.4

a Volume of pores larger than 12 μm.

b Macropores between 12 μm and 50 nm.

c Mesopores between 50 and 5.6 nm.

d Micropores smaller than 5.5 nm.

**Figure 8:**
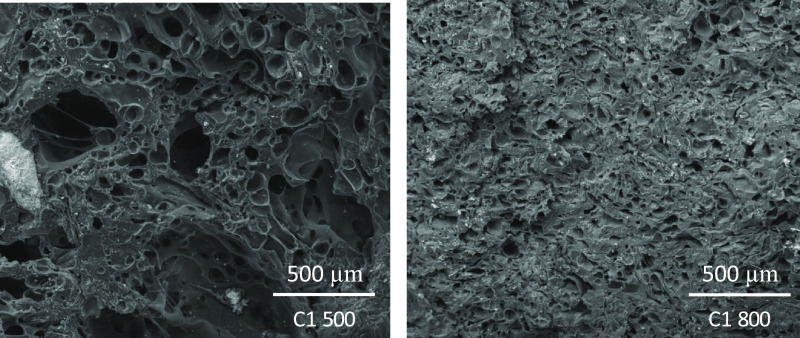
SEM micrographs of C1 semicoke surfaces obtained at 500 and 800 °C.

It is clear that the difference in pore size distribution is caused by changes in temperature. In general, increasing the final temperature produces a higher percentage of micropores and a lower percentage of mesopores. The behavior of suprapores with temperature depends on whether the coal is dangerous or safe. In the case of dangerous coals, the volume of suprapores (>12 μm) is higher in semicokes produced at 500 °C (SK500) than at 800 °C (SK800). This indicates that as the gas has difficulty in escaping, it causes the structure to expand, giving rise to a higher volume of large pores. These results were supported by studies using scanning electron microscopy (SEM). As an example, semicokes from the dangerous coal C1 SK500 and C1 SK800 are shown in Figure 8. Pore size is the most significant difference between the two images. It is clear that the pore diameter of C1 SK500 is larger than the pore diameter of C1 SK800, which shows a reduced pore size, a smoother pore edge, and a denser and more homogeneous surface.

**Figure 9:**
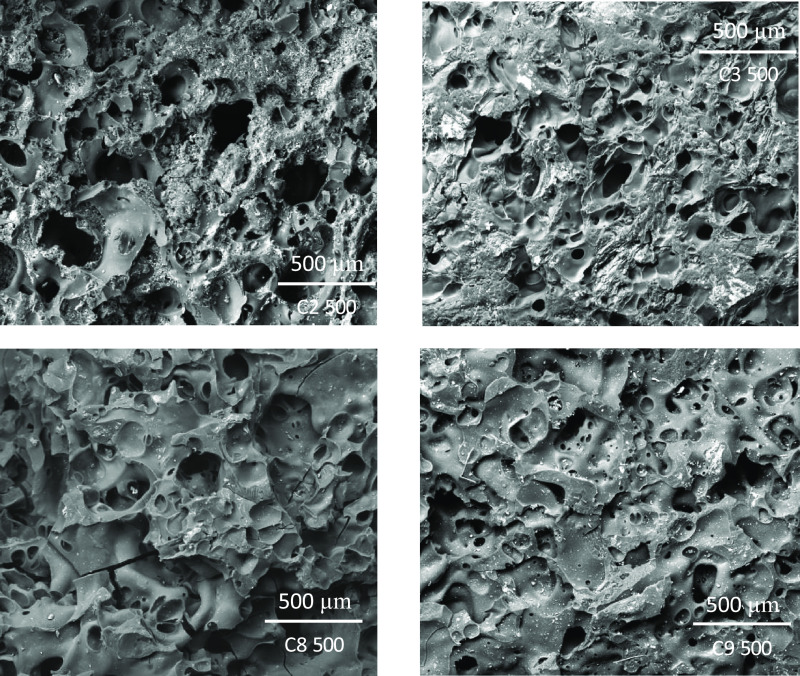
SEM micrographs of semicoke surfaces obtained from dangerous coals C2 and C3 and safe coals C8 and C9 at 500 °C.

**Figure 10:**
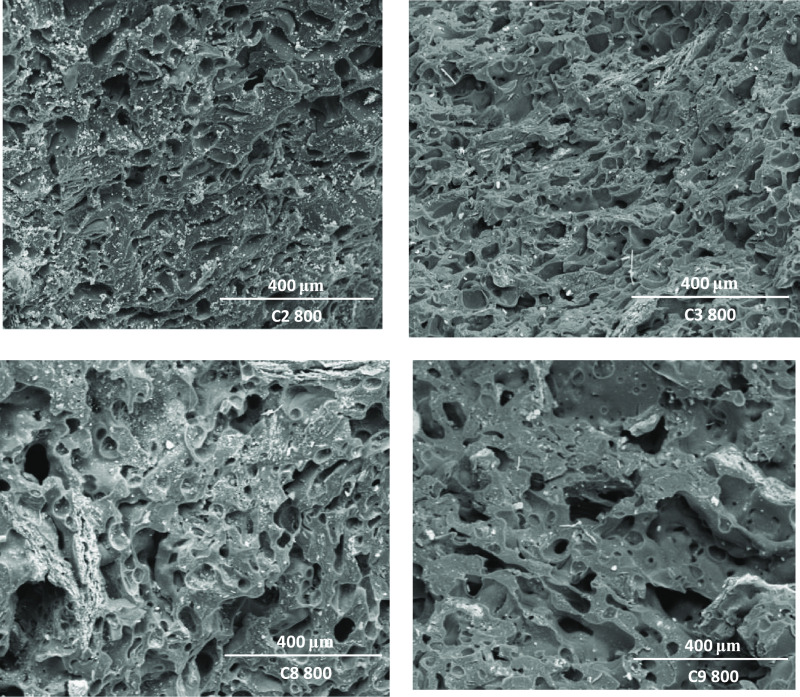
SEM micrographs of semicoke surfaces obtained from dangerous coals C2, C3, and safe coals C8 and C9 at 800 °C.

The pore size distribution for the same temperature differs depending on the dangerousness of the original coal used. Semicokes show a higher percentage of macropores in dangerous coals than in safe coals. In the case of suprapores from SK500, no evident trend was noted on the basis of the data obtained by mercury intrusion porosimetry (Table 7). However, a comparison of SEM images for SK500 shows that the suprapores present in the semicoke surface from dangerous coals shows a larger pore size than the suprapores from safe coals, whereas an opposite trend was noted in the case of SK800. In addition, according to porosity, surface of safe coals looks like low aerated foam for both stages, plastic layer and semicoke structure, while dangerous coals appear like high aerated foam (Figures 9 and 10).

**Figure 11:**
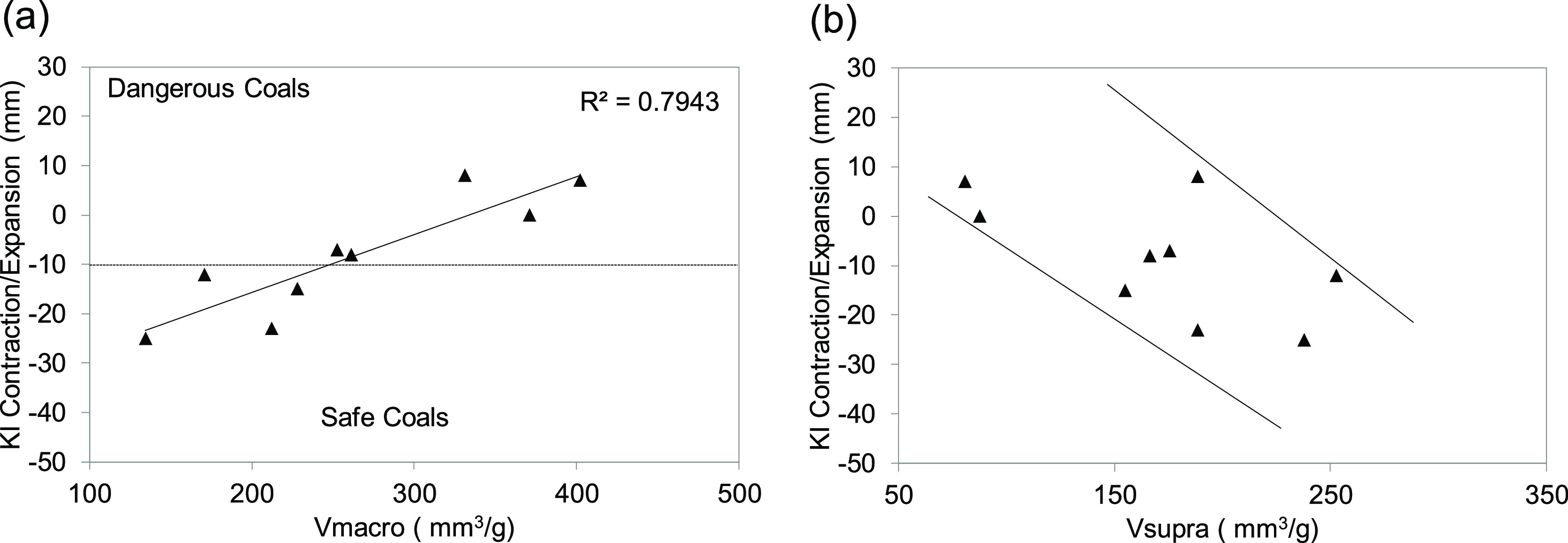
Variation of (a) macropore volume and (b) suprapore volume with Koppers contraction/expansion for semicokes prepared at 800 °C.

The variation in the macropore and suprapore volumes of the semicokes prepared at 800 °C has been plotted against the Koppers contraction/expansion (Figure 11). From these graphs, it can be seen that dangerous coals are associated with higher macropore, whereas in the case of the suprapore volume, the behavior observed was the opposite. Pore formation starts just below the softening point. During this stage, a solid–liquid–gaseous mixture is present. Any volatile matter released produces bubbles that can grow and merge with neighboring bubbles to create larger bubbles. The possibility of the formation of channel-like pore structure at the semicoke side increases. When the balance between the entrapped bubbles in the plastic stage and the surrounding material is broken, the gas is released and pores are formed. In the case of dangerous coals, plasticity is low and the low-permeability layer is maintained intact over a longer period of time than in safe coals. Due to the high viscosity of the medium, bubbles cannot grow, coalesce, or move freely and the connectivity of pores decreases. Therefore, the number of pores larger than 12 μm decreases, while the number of macropores increases.

With mercury porosimetry, it is possible to determine open porosity; however, it remains blind to closed porosity, and therefore the relationships established in this study between porosity and coking pressure refer only to open porosity. Previous studies suggest that the presence of closed porosity in dangerous coals may contribute to a thick low-permeability zone, which may impede the movement of gas in the direction of the oven walls.^22,24^

## 4 Conclusions

To obtain a better understanding of the development of coking pressure during the carbonization process, the coal plastic layer and the semicoke layer of nine coking coals were investigated.

In the case of dangerous coals, the temperature of maximum volatile matter evolution is close to the resolidification temperature, and there is a large low-permeability zone that covers most of the plastic layer and part of the semicoke. Dangerous coals have a higher porosity, a lower Hg apparent density, and a higher macroporosity with a macropore size in the range of 50 nm to 12 μm.

In contrast, safe coals show a low-permeability zone of very short duration, which only affects a small part of the plastic layer. Although semicoke porosity is lower, the presence of suprapores with size between 12 and 250 μm in the SK800 is greater than that in dangerous coals, facilitating the escape of volatile matter and avoiding the buildup of coking pressure.

## Supporting Information Available

Maceral composition (Table S1) and diagram showing the semicoke sampling (Figure S1) (PDF)
