# Supplementary material for: Permeability and Porosity Development during the Carbonization of Coals of Different Coking Pressures
Source: Energy Fuels. 2021 Mar 15;35(7):5808–17. doi: 10.1021/acs.energyfuels.0c04219 (PMC8480075; doi:10.1021/acs.energyfuels.0c04219)
Supplement: Supplementary file 1 — ef0c04219_si_001.pdf [file ef0c04219_si_001.pdf]

Supplementary Material

PERMEABILITY AND POROSITY DEVELOPMENT DURING THE CARBONIZATION OF COALS OF DIFFERENT COKING PRESSURE

M. Dolores Casal, Elvira Díaz-Faes, Carmen Barriocanal*

Instituto de Ciencia y Tecnología del Carbono, INCAR-CSIC, Francisco Pintado Fe, 26 33011 Oviedo. Spain

Experimental

A petrographic examination of the samples was carried out on a MPV II Leitz microscope under reflected white light using immersion objectives (32×) in accordance with the ISO 7404-5 standard for vitrinite reflectance and the ISO 7404-3 procedure to determine the maceral group content.

Table S1. Maceral composition (vol % mineral matter free)

Coal	Origin country	Vitrinite	Liptinite	Inertinite
C1	America	86,1	0,0	13,9
C2	America	83,6	0,2	16,2
C3	America	91,1	0,0	8,8
C4	Australia	82,7	0,0	17,3
C5	America	84,6	2,2	13,2
C6	Australia	77,2	1,4	21,4
C7	Czechoslovakia	56,9	8,1	34,9
C8	Australia	82,2	4,4	13,4
C9	America	73,3	12,2	14,5

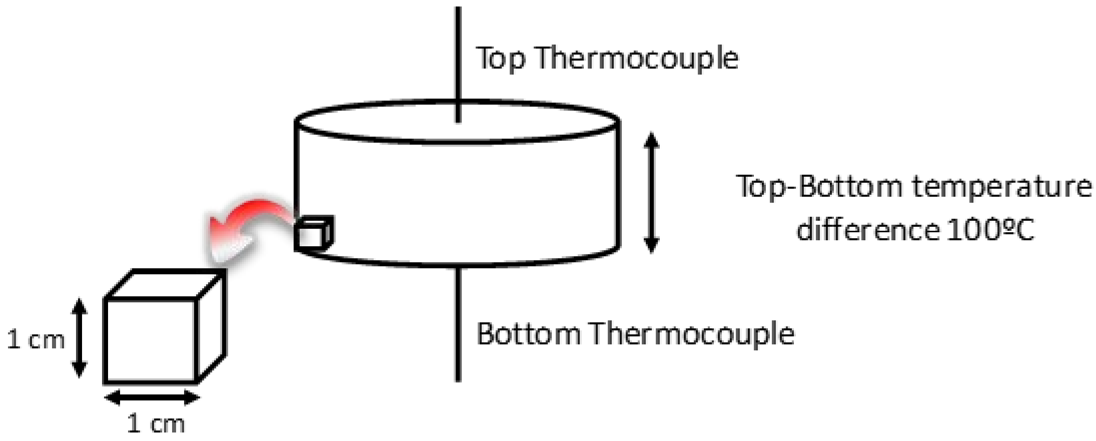

Figure S1. Diagram showing the semicoke sampling.
